# Supplementary material for: Longitudinal Outcomes Following Mitral Valve Repair for Infective Endocarditis
Source: Microorganisms. 2024 Sep 1;12(9):1809. doi: 10.3390/microorganisms12091809 (PMC11434133; doi:10.3390/microorganisms12091809)
Supplement: Supplementary file 1 [file microorganisms-12-01809-s001.zip › microorganisms-3043637-supplementary.pdf]

Supplementary Table S1. Risk factors associated with mortality

Covariate	Hazard Ratio ± Standard Error (*p*-Value)
Age	0.99 ± 0.02 ($p = 0.5$)
Female	1.58 ± 0.73 ($p = 0.3$)
Atrial fibrillation	2.36 ± 1.52 ($p = 0.2$)
